# Lived experiences of closeness to a person using Anabolic androgenic steroids a next of kin perspective

**DOI:** 10.1080/17482631.2023.2292826

**Published:** 2023-12-12

**Authors:** Annica Börjesson, Margaretha Ekebergh, Marja-Liisa Dahl, Lena Ekström, Mikael Lehtihet, Veronica Vicente

**Affiliations:** a Department of Laboratory Medicine, Division of Clinical Pharmacology, Karolinska Institutet, Karolinska University Hospital, Stockholm, Sweden; b Department of Neurobiology, Care Sciences and Society, Karolinska Institutet, Stockholm, Sweden; c Faculty of Caring Science, Worklife and Social Welfare, University of Borås, Borås, Sweden; d Department of Medicine, Karolinska Institutet, Stockholm, Sweden; e The ambulance medical service in Stockholm (AISAB), Academic EMS Stockholm and Department of Clinical Science and Education, Södersjukhuset Karolinska Institutet, Stockholm, Sweden

**Keywords:** Anabolic androgenic steroids, AAS, use, next of kin, relative, phenomenology, reflective lifeworld research, caring science perspective

## Abstract

**Purpose:**

Anabolic androgenic steroids (AAS) are used for their aesthetic and performance-enhancing effects and are associated with physical and psychological side effects. Behavioural changes/side effects as mood swings, aggressiveness, depression, potency problems, anxiety, and emotional coldness have been reported by next of kin to people using AAS.

**Methods:**

This phenomenological study is based on the reflective lifeworld research approach. Interviews were conducted with twelve next of kin about their experiences of living close to persons using AAS.

**Results:**

Next of kin to persons using AAS are particularly vulnerable because they experience little opportunity to influence their situation. Their given and safe context is lost, and their lives are circumscribed by feelings of insecurity, fear, powerlessness, and grief. Feelings of loneliness develop when their problems are not noticed by others and support is lacking from family and society.

**Conclusions:**

Our research adds important knowledge on how the use of AAS affects next of kin. Understanding is required to approach the lifeworld of next of kin with flexibility and empathy in their difficulties and vulnerability. Healthcare professionals and other concerned professions need to be aware of next of kin existential needs to be able to meet and support them in their life situation.

## Introduction

High doses of Anabolic Androgenic Steroids (AAS) increase strength, muscle, and fat-free mass (Bhasin et al., 1996; Rogerson et al., 2007) and are used for aesthetic purposes, being widespread in many countries. It has been proved difficult to estimate the number of users worldwide, however, the use of AAS is considered a public health problem (Kanayama et al., 2018). The great majority of people using AAS are male (Ekström et al., 2021; Pope et al., 2014). AAS are also used by women (Börjesson et al., 2016; Ip et al., 2010) but to a much lesser extent.

Interview studies with men and women using AAS (Börjesson et al., 2021a, 2021b) show that achieving the perfect body involves existential challenges. Men sculpt their bodies to create the perfect physique based on a masculinity norm. Women strive for the perfect body while struggling to maintain the balance between muscle development and acceptable side effects. Body dissatisfaction is mastered by hard training, a strict diet, and the use of AAS. Lack of self-esteem contributes to the experience of the imperfection of the body and is compensated for by self-control, discipline, and performance. AAS are categorized as illegal substances in Sweden because of their health hazards (Riksdagen, 1992). Due to their illegality and society’s attitude towards the use of AAS, people using AAS fear revealing their use to others (Börjesson et al., 2021a, 2021b).

High doses of AAS increase the risk for side effects. Men’s most common reported somatic side effects are decreased libido, gynaecomastia and acne (Börjesson et al., 2020; Sjoqvist et al., 2008) and women’s are clitoral enlargement, voice change, reduced breast size, and increased body hair (Börjesson et al., 2016; Gruber & Pope, 2000; Ip et al., 2010). Research among next of kin to persons using AAS is rare, but side effects have been documented although to a very limited extent (Eklof et al., 2003; Ekström et al., 2021; Havnes et al., 2019). Mood swings, aggressiveness, depression, and emotional coldness are mental and behavioural changes that next of kin experience in persons close to them who use AAS (Havnes et al., 2019). In a study of side effects reported by both persons using AAS and next of kin, the former primarily report physical side effects while the latter mainly report psychological side effects (Ekström et al., 2021). Mental and behavioural changes have also been described previously when next of kin have contacted telephone counselling in Sweden aimed at people who are concerned about or affected by someone’s non-medical use of AAS (Eklof et al., 2003). The most common reasons for next of kin to contact telephone counselling are because they want information about the use of AAS and because they are worried about persons close to them who use AAS. They consider information as important to help them understand the perceived side effects of the substances. They are also in need of counselling because they are personally affected by the behaviour of persons using AAS. In addition to the side effects mentioned above, persons using AAS are perceived as absent-minded, antisocial, lacking in empathy and sometimes even frightening (Ekström et al., 2021). Next of kin have also said that they need personal advice on how to act and react to AAS use, and on the kind of help available to the family in a difficult situation (Eklof et al., 2003; Ekström et al., 2021).

Research has been carried out on people who use AAS, but the scientific literature on next of kin experiences is almost non-existent. As described above, next of kin are often deeply affected by someone’s use of AAS, and therefore it has been important in this study to highlight their existence and experience. This study aims to deepen knowledge and understanding of next of kin’s lived experiences of closeness to persons using AAS.

## Materials and methods

### Design

This phenomenological study is based on a reflective lifeworld research (RLR) approach (Dahlberg et al., 2008). The phenomenon being explored and illuminated in this study is next of kin’s experiences of living close to persons using AAS for non-medical reasons. The lifeworld perspective helps us to seek understanding in the lived everyday world. The lifeworld is the ordinary world that is shared with other people, but it is also our unique existential world as it is experienced by each human being. The world is perceived through our natural attitude, so it is necessary to be aware of this to understand the phenomenon itself. Many things are taken for granted or in other words are not reflected upon, and therefore researchers need to keep their distance to this natural attitude. Important methodological principles in RLR are openness, flexibility and bridling. Bridling strengthens the openness through the researcher’s reflective attitude and flexibility towards the phenomenon. By reflection, we can raise awareness and conceptualize the lifeworld. We have been objective in a phenomenological sense in which personal values, theories and other assumptions may impede us from acquiring a new understanding of meaning (van Wijngaarden et al., 2017).

### Study setting

In this study, next of kin are defined as people who have close relationships with persons who use AAS. Partners, parents, and siblings have been included. This definition is based on the fact that these people, who describe close relationships with persons using AAS, are the most frequent callers to the Anti-Doping Hot-Line in Sweden. To participate, next of kin had to understand the Swedish language and to be over 18 years of age.

The participants were recruited in two ways: either by contact with Anti-Doping Hot-Line (*n* = 10) or via snowball sampling (*n* = 2). The Anti-Doping Hot-Line is an anonymous free telephone counselling service started in 1993 for people concerned or affected by the non-medical use of AAS (Eklof et al., 2003). The Anti-Doping Hot-Line is localized at the Department of Clinical Pharmacology, Karolinska University Hospital in Stockholm, Sweden. It is managed by trained nurses and clinical pharmacologists. The recruitment of study participants via the Anti-Doping Hot-Line was done by two registered nurses working in counselling, one of whom is the first author of this study. The callers were asked if they were interested in participating in the study after the counselling session. If they were interested, the participants received oral information about the study. In the next step, written information was sent by email. After the participants reconsidered their participation in the study, they reconnected via email if they agreed to participate.

The recruitment via the Anti-Doping Hot-Line was not quite adequate to be able to describe all the nuances and variations of the phenomenon. Therefore, two informants were recruited via snowball sampling. Contact was made with an initial person who conveyed contact. Oral and written information were received in the same way as described above. Snowball sampling is a convenient method of reaching hard-to-reach and hidden populations in order to study social network structures (Heckathorn, 2011).

### Participants

In this study twelve next of kin were included, of whom nine were partners/girlfriends/wives, two were mothers and one was a sibling. Most of the interviewees were women (*n* = 10). Each of the 12 participants was closely related to a man (*n* = 10) or a woman (*n* = 2) with current or previous use of AAS. For all except one of the participants the use of AAS had been ongoing for a longer period (several years). Nine of the persons currently used AAS and three had previous use. Eight of the interviewees lived in the same household as the person who used/had used AAS at the time of the interview (two of them no longer used AAS). The other four next of kin had either been separated or the person using AAS had moved away from home. Seven out of eight partners lived together with children. Four of them have or had been in contact with social services because of an unsustainable situation due to their family’s insecurity and unrest. One of the separated partners was involved in a child custody dispute.

### Data collection

The lifeworld interviews lasted 45–120 minutes and were conducted by the first author AB. The interviews took place in an undisturbed setting (separate room) in a library, at the university or in the interviewee’s home. Two of the interviews took place by telephone based on the informants’ wishes. As all the informants lived spread out across the country, the interview might take place within a couple of days to a couple of weeks after approval of participation in the study. The interviews began with the opening question: Can you tell me what it is like to live close to a person who uses AAS? To be able to support the informant in clarifying, deepening, and explaining what was meant and to encourage detailed descriptions of the phenomenon, follow-up questions were asked (e.g., how do you mean, can you describe more?). The interviews were recorded in their entirety on audiotape and transcribed verbatim.

### Data analysis

The guidelines for the RLR analysis—openness, bridling and flexibility—were followed during the whole analysis. To create a deep feeling for and understanding of the whole text, the analysis process started with open-minded, repeated readings of the interview transcripts and their meanings. During the initial reading the researchers let go of their natural unreflective attitude and adopted an attitude of carefulness and reflection during the analysis. Nothing was taken for granted and meanings were questioned and pondered upon. The researchers were careful and slowed down the process of understanding so as not to be too quick to make definite what was indefinite (Dahlberg & Dahlberg, 2003). When the data was familiar as a whole, it was divided into smaller parts in search of meanings followed by a movement between the whole (interviews) and the parts (meanings of the data). Reflection and questioning were repeated until meanings could be clustered together, and the meaning of the phenomenon slowly began to emerge from the pattern between the clusters. When the phenomenon become visible, a new whole was reconstructed (essential meaning) (Dahlberg et al., 2008). In the analysis process, the focus is on the phenomenon and not on the individual. The aim is to search for meanings in all experiences collectively.

All authors in this study have different experiences of the topic AAS. The first author (AB) has extensive experience in providing information and support regarding side effects for people concerned or affected by the non-medical use of AAS. Authors LE, ML and MLD have extensive knowledge and experience of research in the field of anti-doping and/or side effects of AAS. ML has many years of clinical experience treating individuals for AAS related side effects. Authors ME and VV have extensive methodological knowledge of reflective life-world research and experience of in-depth interview technique from a life-world perspective. On two previous occasions, all the authors have together carried out reflective life-world research within the topic of AAS.

To ensure quality and transparency, a checklist (COREQ32) for reporting qualitative research were used.

### Ethical considerations

The informants were informed that participation was voluntary and of their right to withdraw their participation at any time without explanation. They received oral and written information about the aim of the study before and at the time of the interview and about the confidentiality of the interviews before giving their written consent. Ethical approval was obtained from the Regional Ethics Committee in Stockholm (no. 2016/1762–31/5). Participants were offered support if they so desired after the interview.

## Results

The meaning of the phenomenon is first presented by the essential meanings, followed by individual meanings.

Living in a close relationship with a person using AAS means living a life that is largely dominated by their interests and needs, which means that one’s own interests and needs may be subordinated to them or ignored. The users’ selfish behaviour on the other hand is made light of or denied, and a tactical and flexible adaptation takes place in the next of kins’ behaviour to fit into an existence with restricted freedom. This oppressed existence is fraught with loneliness, fear, and insecurity as well as a feeling of being betrayed and not having any support. However, it is a suffering that must be endured because next of kin lack the courage to face revealing to others what life is like. Feelings of shame and powerlessness develop, and they are handled by means of concealment or possibly even defence of the behaviour of persons using AAS. Despite the fact that this existence breeds anger and aggression towards persons using AAS, next of kin feel powerless to change the situation. They feel sympathy for them and nourish ideas of being able to help and protect them. Next of kin’s self-esteem and confidence in their own ability to act fail because of repeated damage to them. Since persons using AAS are in a “bubble” in which only their own needs and self-centred behaviour are in focus, next of kin become invisible. Not being able to reach persons using AAS in a dialogue causes sadness and anxiety. This whole unmanageable situation may contribute to social isolation in order not to have to reveal what life with persons using AAS is really like. When the suffering becomes unbearable, the grip on keeping up appearances is released, and life is at stake to make persons using AAS break their behaviour.

### Adaptation to an existence with restricted freedom

It has been shown that next of kin’s relationship to persons using AAS changes and becomes emotionally cold, hard, and loveless, though formerly characterized by closeness, love and caring. The understanding and consideration that previously existed towards the family may cease. Persons using AAS do not show affection and are experienced as indifferent. There are no hugs or body contact, and partners do not feel seen. Adapting to a changed relationship that is perceived as loveless affects next of kin’s freedom to live their lives as usual. They feel sad about no longer having close contact, not being able to share experiences and not having an everyday life together. Intimacy and sex life change, with the sex drive varying according to the timing of AAS use. Partners experience that the sex drive of persons using AAS can at times be very demanding while during other periods it is completely non-existent: *“I could do like anything at all, but it was still totally uninteresting. And that was bloody tough because I still have needs, so how were we going to solve this”*. Lack of sex drive is something persons using AAS prefer to avoid talking about as a problem and therefore it is swept under the carpet. When it is brought up, they lie or blame others and insist that it is not their fault, which leads to partners easily coming to believe that this problem is caused by them.

Next of kin are forced to adapt their lives to the time-consuming needs, lifestyle and routines of persons using AAS, whose main focus is on diet and exercise: *“His only focus was on himself, all the time, he had an eat and sleep clock and it was like exercising and this and that … he was the only person in existence”*. Partners describe taking full responsibility for the children on their own, all meals, activities, and household chores, which is burdensome. Persons using AAS are not mentally present or involved in their families’ activities and commitments. Running an entire household alone contributes to there not being enough time, and concern develops that the children’s needs are not being met. This time-constrained situation also restricts the freedom to meet one’s own needs and interests. The kind of life led before can no longer be lived. An unexpected occurrence may lead to consequences. One next of kin says:

I contacted him: ‘you must pick up the children, I’m in hospital’. ‘No, I don’t have time for that’ or ‘I can’t afford to pay for that’. ‘I’m very sick, I can’t pick them up, I have no one to help me. You have to hurry to pick up your children, they’re at school’. But he did not. So I actually had to ask them to pull out the infusion and then I had to go and pick up the children and leave them with a friend. Afterwards I went back to the hospital. This was quite risky for me, because it was so serious.

The use of AAS may also affect the family’s finances. Persons using AAS do not always contribute their share to the household, as their money is needed for their own expenses. The shortage of money leads to stress that there will not be enough for the most basic needs. The opportunity to live a freer life is limited because next of kin cannot choose what they want to use their own money for: *“It was tough running everything on your own. Yes, it was. Running a company on your own and responsibility for three children. No sickness compensation or anything. I couldn’t take a holiday, I had to work”.*

This existence with restricted freedom becomes manifest when next of kin are compelled to distance themselves socially. Meetings with family and friends are avoided. In addition to not having enough time, the distancing is also due to the strict eating rules of persons using AAS. Inability to eat the food offered constitutes a hindrance to being invited to dinner or eating out at a restaurant/café where usually the right kind of food is not available. One partner relates:*“We can’t go out and eat spontaneously or anything. It affects a lot of things because I am really a social person”.*

### Mood swings create an insecure existence

The dominating behaviour of persons using AAS controls and influences their partners’ existence. Partners want everyday life to function smoothly and therefore adapt to this behaviour, thus creating a better atmosphere in daily life. Partners describe that mood swings, which can be very powerful, are related to the time of the AAS use. There are good and bad periods. One woman says: *“And these outbursts. It’s like a record going round and round. Every time we have a good period, I know that I can’t enjoy it, because I know that there will soon be a bad period again”.*

The atmosphere in a room may change very quickly and the large body size of persons using AAS can cause fear and anxiety for outbreaks of violence. Persons using AAS will normally direct their anger at the person’s closest to them, usually those who “support him through fair and foul”. To try and prevent all too heavy and violent mood swings, next of kin “tiptoe around”, attempting to interpret and sense the atmosphere. They try to read the persons using AAS to prepare themselves for different situations. This is a tactic they have developed to avoid confrontation and unpleasant situations: *“I sense him almost immediately when I meet him, what mood he is in, so that I can put myself in a ready position somehow”*. Through this adaptation, the mood swings of persons using AAS become less unstable, and partners may be able to prepare better for when the mood fluctuates again.

However, next of kin do sometimes try to confront by pointing out which side effects they notice and experience, and they do occasionally express their dissatisfaction. They also try to discuss how the use of AAS affects both the persons using AAS and the entire family and they also attempt suggesting how to put an end to this use. This kind of confrontation is mostly met with a huge portion of irritation and aggression by persons using AAS, who state that the image being painted is completely incorrect. Persons using AAS often claim that their next of kin have no knowledge about AAS, unlike themselves. They perceive themselves as experts in the field:

And when he says that everything regarding his condition is my fault, then I bring up the substances that he uses and what the side effects are, although he claims that he has no side effects. He gets no side effects. It has nothing to do with that. And he thinks that I should not say anything about it because I know nothing.

Persons using AAS also often tell next of kin that the situation they are experiencing is not due to AAS, instead it is due to the partners’ own faults and shortcomings. Partners may also be subjected to various accusations, such as condescending comments about their bodies not being perfect enough:

You’d have been very good-looking if you’d had surgery to remove that skin on your stomach and you’d have been really good-looking if you’d had a boob job done, then you’d have got 10 out of 10 as well.

Furthermore, persons using AAS often accuse their partners of not focusing on or prioritizing routines around food and exercise, for their inability to perform tasks in the right way, or even for not being easy to live with:

He gets really angry. For the smallest thing. And you cannot talk to him. He’s got megalomania, I’d say. He makes me feel very small, everything I say is wrong. And he makes me back off more and more since it doesn’t matter what I say.

In summary, the behaviour of persons using AAS contributes to their next of kin’s feelings of being hurt, oppressed and inadequate. The feeling of being useless is reinforced by the fact that persons using AAS do not apologize for their behaviour. Their self-preoccupation is deeply hurtful. Next of kin’s fear of being exposed to threats and violence makes them adapt to the will and opinion of persons using AAS and not question, criticize, or confront unnecessarily. Instead, they maintain an attitude of “agreeing” or being silent.

Children in close relationships may also be exposed to verbal, physical and psychological abuse due to the rapid mood swings of persons using AAS. Partners may worry about their children’s mental state as they are not allowed to play and be loud or disturbing, which is normal behaviour for children. To make children obey, threats and violence may be used. In this way, persons using AAS show who makes the decisions in their family:

He just lashes out verbally “stupid, are you?” or grabs his chin, or just gets angry if he spills a drink, he gets angry if he happens to wet himself, doesn’t make it to the toilet in time or like, “are you a baby, hey?”. He is –, in his world it’s completely normal and I don’t think that’s the way to behave”.

Children exposed to this violence have to live in constant fear of doing something wrong. Because of this they withdraw into themselves and change their behaviour to try to please:

They love both their parents, but of course I do notice that it really affects him because … he gets so scared. It was not long ago at all that we were washing ourselves before putting him to sleep, and there was water on the bathroom floor. And he started crying and told me to hurry up and wipe the floor “before dad sees it because then he gets angry and swears”. Well, obviously this affects him. He often says, “otherwise dad gets angry.

Partners are aware of the risk that the children may not feel comfortable or that their needs will not be met if they are left alone with persons using AAS. This leads to fear of leaving children alone with them.

### Meeting the need for confirmation

Next of kin feel that it is not possible for them to satisfy the needs of persons using AAS for constant validation of their appearance. The search for approval from others is constant. Next of kin believe that a life history including a troubled upbringing, bullying, lack of affirmation during childhood, and low self-esteem may have caused this great need for approval in persons using AAS. It does not get any easier when other people comment on a built-up body and show their admiration. One next of kin says:

He gets comments you know – ‘Crikey, you’re just so big and handsome’. So for him, it’s an incredible boost for his self-confidence, I imagine that he must feel really good about it.

Muscular bodies and comments from others increase the self-esteem of persons using AAS. This may lead them to show off their bodies on social media. There, other people show them respect for their success and performance, as well as the appearance and size of their muscular bodies. One partner describes her experience:

He started posting accounts, with hundreds of naked pictures of himself. He shows off his body and says straight out “I need to be affirmed, I want this, this is important to me and you have to respect that”. The self-validation is huge, it’s the only thing, it’s the only thing in life. The need for affirmation – ‘see me, see me, see how big I am, how different I am compared to everyone else and so on, that’s what the whole thing is about.

The need for affirmation and self-centred behaviour—the constant search for approval and the all-prevailing self-centredness—of persons using AAS also create feelings of intense annoyance. Two partners comment: “*If there was a mirror there, then he was in front of it”; “If we walked past a mirror, a windshield or the window of a store, he spent all his time searching for and looking at himself”.*

Self-validation was sought from the people around, even in front of a partner. One partner says:

I might be only two metres away when I see that he – for one thing she looked pretty – that he was searching for her so that he could see if she was paying any attention to him and in that case, this was an affirmation of him looking handsome.

Next of kin feel shame in front of other people about how persons using AAS post pictures of their bodies on social media, showing themselves naked in front of other people. They do not want to be associated with someone who stands out in this way from the crowd. They are also worried about what other people will think because it is obvious that such bodies have not been built in a natural way, and this kind of behaviour is not in line with respectable parenthood. Next of kin to female users of AAS also feel ashamed that they have developed masculine features:

This is about our older relatives, our uncles who can also see this kind of picture. She stands out from the crowd in a way that we do not think is positive. Both when it comes to exercising and pictures and so on.

Each whole family is naturally affected by all the external affirmation, which unfortunately reinforces the extreme need for attention of anybody using AAS. These persons’ behaviour and lifestyle are constantly highlighted and prioritized, which makes their families feel uncomfortable. However, people outside the family are not aware of what the use of AAS can cause. Affirmation from other people encourages the continued use of AAS which affects the whole family’s life situation. One partner describes this:

What annoys me is that society responds to and confirms him. Like I said before people think he’s so talented. No, he’s not that at all, you have no idea. No one knows a shit, they don’t understand anything. If he had been an alcoholic then people would have said –‘oh poor her, how is she coping, living with this’. They would have agreed with me. Now all I get is – ‘yes she left him, the guy that’s so awesome, how can she leave him, he’s so cool, look how big he is’”. “You really aren’t smart, but it’s what’s behind it as I said, you have no idea how we feel back here.

There are clearly reasons why AAS are largely used in secrecy. Next of kin do not want other people to know their actual situation and they also find it difficult to talk about. They feel shame when telling relatives, friends, and acquaintances that persons they live with use illicit drugs. Partners may feel that others perceive their living with substance abusers as failure, due to society’s negative opinions and preconceptions about individuals with substance abuse. Partners worry that their children’s friends’ parents will not allow their kids to play together. This makes partners try to keep persons using AAS out of others’ sight. They avoid talking about them, their behaviour or their changed body size. One partner says frankly:

I’ve hidden him away. I’ve kind of not talked about him and told others that he can’t come, I’ve made excuses about him having to work overtime. In the end, if he posted posts that were revealing, I made those posts not visible. I do not want to be associated with that and that is probably why I’ve hidden everything all the time as well. I don’t find it fun when people see it, like when you have to be responsible for other people’s children. Just think how they think, will they dare to send their children home to us, thinking of him being there and how he is behaving, what kind of people are we … so you feel very ashamed.

### Inability to leave the relationship

Persons using AAS do not listen to any demands that they must stop using AAS, leaving next of kin feeling abandoned and frustrated. Next of kin’s attempts to discuss the risks therefore become increasingly half-hearted. Besides that, it is difficult to have this discussion because they are afraid that persons using AAS will become threatening and aggressive. It may also be the case that next of kin do not dare to confront them for other reasons such as not having adequate arguments and knowledge. However, they gather information continuously in order to prepare for the right opportunity for a dialogue. Next of kin sometimes try to threaten to leave the relationship if AAS use is not terminated, but without success. Losing one’s family is not sufficient reason to end the use of AAS:

His love for these substances is greater than his love for me.

Persons using AAS show clearly that they do not appreciate being confronted or subjected to negative criticism:

As soon as I want to talk about something, he raises his voice and gets angry. He shouts and he can be perceived as threatening. I’m afraid he’s going to hurt me.

Despite the emotionally cold and unempathetic approach of people who use AAS, next of kin feel concern for them and therefore it is difficult for them to leave their relationships. They choose to stay anyway and strive towards family normality. This means that they endure humiliation and remain hopeful about change in their relationship. Sometimes, however, next of kin may succeed in achieving change by making demands:

I told him I’m taking the kids away from you. That’s when he agreed to treatment. So I said ‘you can do whatever you want after the treatment, but you have to do this to keep the children’.

Persons using AAS may be perceived as constantly absent by other people and family members because they do not participate in family gatherings such as birthdays and other holidays. For the next of kin, it feels difficult that no one in their circle talks about the person using AAS anymore, despite this person being constantly present in their lives and awareness.

Family members such as parents and siblings dream that one day they will be able to spend time together again as before, although this would require insight in persons using AAS about their need for help. Parents who have been rejected describe how difficult it is to accept that they can no longer be involved in their adult child’s life. It is hard to let go of someone you love. They cannot abandon or leave their child, instead they put their own lives on hold to be constantly on standby if the need for assistance should arise. This constant availability can be demanding and long-lasting. One mother recounts:

I felt that now somewhere I have to give up. He’s in this and it will never change until he’s stopped taking anabolic steroids. I cannot make him stop. Now I must try to be more involved with my daughter and my grandchildren instead.

Grief is a strong emotion in these parents who are excluded from their children’s lives. They feel they are losing their child. They also feel fear of not knowing if something is happening, or as one mother puts it:

So I feel that either way he dies, like. He’s been in hospital twice with heart problems and that is probably the biggest concern I have, what if they don’t even contact me when he is in hospital?.

### Need for support

Next of kin often seek support from their immediate family such as their parents-in-law or their partners’ siblings when they perceive the situation as untenable. They ask for help to communicate with their partners. However, it often turns out that families deny the existence of any problems, often due to their fear of the moods and large bodies of persons using AAS. They also prefer to not get involved in anything that could lead to a conflict. This lack of support within one’s own circle leads to seeking information and support from various authorities such as the police, municipality, social services, a psychologist or psychiatry. However, the authorities often lack the knowledge, tools, and resources to be able to help the families of persons using AAS. They do not want to deal with AAS use. The authorities shut their eyes to these problems because they fear confronting and making demands on persons using AAS. Their fear is based on these persons’ body size, and their view that all persons using AAS are aggressive. One woman recounts her first meeting with social services staff: *“And then he says, I think we have to take things a little carefully because he is so very big, it can be a little scary so I think that we have to be careful, I don’t think we should ask him to leave samples, I think we must be careful”.*

According to next of kin, schools may report their children’s troubled condition to the social services. Next of kin would have wanted the social services to take this much more seriously since children may be terrified by the behaviour of persons using AAS. Next of kin feel that parents’ rights have priority over their children’s rights and best interests. This means that children may be compelled to meet their parent even though they do not feel at ease about it. One woman says:

So it is a disaster that the social services don’t dare to tackle this part of the problem. Because aren’t they there to help children to a better life in the society? Then they need to have the courage to take hold of things that are uncomfortable, otherwise we wouldn’t have been there. I mean the social services are there for uncomfortable situations, you don’t go to the social services when everything is good.

That next of kin need to talk about their problems with someone is obvious. They describe needing to converse with someone they trust, in a similar situation. However, they do not know how to find others in a similar situation and there are no organized groups for next of kin to individuals using AAS. They lack support from their closest friends, family and also society, leading to loneliness and feeling let down. Lack of support compels them to manage alone, not collapse but stand strong for the sake of the children especially.

AAS use can continue for a very long time without anyone noticing that anything is wrong. Most people are not aware of the external signs of AAS use. They perceive persons using AAS as healthy and as taking good care of their bodies. They encourage their physical development, thus triggering further use of AAS according to next of kin:

After all, society makes it possible to continue doing this to me and to the children. Actually. He gets affirmation. He is disciplined, gets pats and praise, which is kind of praise for hurting other people. And that, that’s probably the biggest problem in the whole business. That’s what really keeps them going. After all, they get approval from society to continue with their shit.

However, next of kin, who witness how side effects gradually lead to destructive changes, live with constant anxiety. One of the causes of their concern is about not daring to hand over responsibility for the children to persons using AAS: *“I still do not want to leave my son alone with him”*. It is difficult for children to understand why their parent is no longer part of their life. It is not easy to explain why a parent is not available, does not act as before or does not put their needs first. One partner recounts:

Yes, it would have been better if he had died or something. It would have felt easier to deal with, for me and the children, if he’d been dead, because then he would have been gone with an absence that you can understand. Now it’s hell trying to help the children through something that is totally incomprehensible to them; why has our dad disappeared, why doesn’t our dad want us anymore? He doesn’t even listen to their needs or what they want, or anything, he doesn’t respond to them at all. How can you do that to children who ask for help, or who beg and shout for their dad be there for them? ‘No, I haven’t got time, I’m going to the gym’.

## Discussion

This study has shown that next of kin struggle to cope with stressful life situations. Their lives demand mastering several difficult components, including vigilance and constant availability to persons using AAS. This limits their own freedom, since there is not enough space for them to do what they want or need to do. Their lives have been turned upside down, they are threatened, and they live with constant disturbances and anxiety. Similarities can be seen to next of kin to individuals with drug abuse. They too experience constant anxiety, with the drug user occupying most of their time and thoughts (Richert et al., 2018). Due to their own anxiety and fear, their need for control takes over which means that they set aside, overlook and neglect their own needs (Jackson & Mannix, 2003). They are constantly on standby in case something should happen (Richert et al., 2018) with the aim of helping and protecting. Family members’ adaption to persons using AAS is similar to the behaviour seen in co-dependency. Co-dependency is a controversial and complex term (Bacon, 2014). However, as with next of kin in this study, it is about family members adapting themselves and their behaviour to individuals they are living close to, whose problematic abuse they hope to change. They believe that they may rescue these individuals from their destructive behaviour (Knudson & Terell, 2012).

Next of kin describe feeling ashamed of their life situation, as do next of kin to individuals with drug addiction (Richert et al., 2018). They all struggle against shame and humiliation for not being happy. This suffering undermines their dignity (Eriksson, 2015), leading to avoidance of talking to others about their situation and the behaviour of persons using AAS. They therefore even renounce the companionship of people they are close to. Next of kin to individuals with drug addiction similarly describe wanting to keep their situation hidden from others (Richert et al., 2018, 2021; Rotunda et al., 2004; Weimand et al., 2020).

People who use AAS disguise what life is really like and next of kin adapt their behaviour to reduce or prevent the risk of being exposed to threats and violence. When people can no longer take care of their most basic needs however, assistance is needed from those closest (Eriksson, 2015). Their endeavours to regain security cause next of kin to seek help in order to meet their most fundamental needs and to eliminate current threats. Next of kin in this study received no spontaneous help from the outside. People around them did not see, react, or understand their actual situation, whether family members, the community or the persons using AAS. Next of kin are always in an extremely vulnerable situation, since the people using AAS, other family members and other people around neither confirm nor understand their difficulties. Others also fear daring to act or intervene out of anxiety for aggressiveness and violence. Not getting help and not being able to do anything about their situation increase feelings of vulnerability (Dahlberg & Segesten, 2010). When no one notices or understands the situation experienced, then pain and suffering develop (Dahlberg & Segesten, 2010; Eriksson, 2015). The suffering in their lives includes all aspects of human existence (Eriksson, 2015; Wiklund Gustin, 2003). Not being seen, heard, understood, or loved means lifelong suffering (Eriksson, 1993, 2015).

Next of kin suffer from feelings of loneliness when people they are close to are no longer there to support them (Dahlberg, 2007). The phenomenon of loneliness belongs to human life and, to existence (Dahlberg, 2007) in different periods of life. Loneliness does not have to be of negative nature if it is self-chosen, but next of kin in this study experience loneliness in the grief of what they are about to lose or have lost, i.e., their loving and trustful relationship when their partners prefer AAS to them. They have been chosen and yet abandoned by the using person for AAS. They miss the vital togetherness they experienced previously, for which they now long. They have also lost their social life with other people close to them. All these losses cause loneliness, insecurity and depression (Dahlberg & Segesten, 2010).

The feeling of loneliness, in this study, may be understood through the concept of existential contexts (Merleau-Ponty, 1945/2013). According to Merleau-Ponty, we humans always belong to a context which we share with other people. We experience the world as a kind of “coexistence” between other people and ourselves. This means that we always have something in common with other people. We share our experiences through a dialogue with others in our social sphere. Our context creates balance and safety in our lives and gives meaning to life. It is fundamental to wellbeing (Dahlberg & Segesten, 2010). For next of kin in this study, opportunities are lacking to share their feelings and experiences with others, as they could in the past. They cannot share their experiences with the persons using AAS for fear of provoking aggressive reactions. They cannot discuss their situation with other people close to them either, as these are not aware of problems caused by living with persons using AAS. In other words, their situation is invisible, and they dare not reveal it to other people since they want to protect their partner or child and they even feel shame for the whole situation. As a result, they do not know how to handle their changed context, being completely alone, lost, and insecure with their feelings and experiences. They have no support in creating a new context. In a metaphorical sense, the people around them only hear “the strong and dominant voice” of the persons using AAS, while their own voice “is weak and ignored”. Thus, they feel disregarded and lonely, feelings which are heavy to bear and difficult to deal with. They are forced to harbour feelings and experiences in their lived body which create dissatisfaction, dejection, powerlessness and illness.

Next of kin in this study experience their suffering as hopeless. They question the meaning of lives and of their grief and powerlessness (Eriksson, 2015). They have to deal with and relate to the suffering of not finding any meaning. According to Frankl, we can cope with great stress if it has any meaning for us (Frankl, 1946/1986). However, when next of kin cannot manage great strain and when attempts to influence and change their life situation do not work, they experience feelings of powerlessness and grief. This are similarly experienced and described by next of kin to individuals with drug addiction when nothing they do or say matters (Richert et al., 2021). Revolutionary incidents can help people to gain new power and take charge of their life situation and change direction (Dahlberg & Segesten, 2010), but this may be difficult when everyday life is ongoing and when they themselves have not been warned about the situation they now find themselves in.

Even though they feel anger and aggressiveness against persons using AAS, next of kin often lack the power and energy to change their situation. This may be one reason why they cannot leave the relationship and therefore endure it for a very long time. Their adjustment to their life situation affects their well-being (Dahlberg & Segesten, 2010). Depression, anxiety, and stress have been shown to be clearly linked with co-dependency (Marks et al., 2012). The lack of the right kind of help from various authorities creates stress which may lead to ill health (Dahlberg & Segesten, 2010; Richert et al., 2018).

Next of kin need to gain the courage to talk about their experiences, to manage their loneliness and create meaning in their suffering. They must make their living situation visible. Only then can they receive the support that is lacking. In the interviews in this study, several informants stated that it felt liberating and good to be able to talk about their situation, which they have been unable to do before. Developing knowledge about next of kin’s need of support is extremely important to prevent their ill health and enable well-being. Next of kin do not share the world of persons using AAS but live in its shadow. From a health and social perspective, this is a group that “the spotlight should be directed towards”, as the results of this study clearly highlight.

## Strengths and limitations

In this study, the informants are predominantly women. This is also common in interview studies with next of kin to alcohol or drug abusers. When it comes to next of kin to persons using AAS, this uneven gender distribution may be partly because most individuals using AAS are men. It is also more common for women to contact telephone counselling regarding AAS use (Eklof et al., 2003). An effort has been made to apply a gender perspective to the next of kin.

The recruitment process has taken a lot of time. This has depended partly on the difficulty of recruiting informants because of their fear of it reaching the ears of persons using AAS and what this would cause. In Sweden, the possession and distribution of AAS are illegal, as is the presence of AAS in the body. Breaking the law may result in fines or imprisonment and a criminal label. Next of kin may thus be afraid to be associated with persons who use AAS or keep/store substances in their shared home. All except two of the study participants contacted the Anti-Doping Hot-Line themselves. Why next of kin contact the Anti-Doping Hot-Line differs, some have general questions about the use of AAS but most of them need personal advice (Ekström et al., 2021). If this study had included other informants, who did not themselves contact the Anti-Doping Hot-Line or needed personal counselling, this may have resulted in other meanings. Therefore, the results cannot be considered representative and transferable to all next of kin of people using AAS. For various reasons, people often hide the truth of their use of AAS (Börjesson et al., 2021a, 2021b). It may be that next of kin are not aware of the use of AAS or they do not experience any signs or problems in their life situation. But it may also be that they are afraid to contact someone because the use is illegal or because they do not want others to know about the use of AAS. Perhaps they feel ashamed of their situation or afraid of the person using AAS. Research has not shown a clear connection between the use of AAS and aggression, but still, it is the most reported side effect over the years by next of kin to the Anti-Doping Hot-Line (Eklof et al., 2003; Ekström et al., 2021). The Anti-Doping Hot-Line is easily accessible, managed by healthcare professionals and the callers can remain anonymous. We believe that these three things facilitated our recruitment process and that this was the most suitable way for us to recruit informants to the study.

The first author in this study has experience of working as a nurse for 15 years in telephone counselling with people concerned or affected by the non-medical use of AAS. The other authors also have varying experience regarding the topic. However, no one apart from the first author has experience of contact with next of kin to people using AAS. To handle the authors preunderstanding, especially the first author, RLR´s principles (openness, flexibility, and bridling) have been followed throughout all research activities. The first author´s experiences have been both an asset and a difficulty. One asset has been familiarity with talking to next of kin in difficult situations and being able to ask the right directed follow-up questions during the interviews. At the same time, it has been a difficulty as things may be taken for granted. A bridling attitude has helped to encounter data in an open manner, not to understand things too quickly and to approach the phenomenon as it is lived and experienced by the informants. The analysis was also carried out in the approach of bridling. The authors adopted an attitude of carefulness and reflection to keep the preunderstanding in check. All descriptions of meaning were questioned and pondered upon because we did not want to force meanings to appear or fall into the trap of seeing what we wished to see. We slowed down our understanding of the meaning and the phenomenon as we wanted the indefiniteness to last as long as possible.

Most of the informants were recruited during a counselling call to Anti-Doping Hot-Line. This means that the interviewer or her nursing colleague (not an author in this study) had contact before the informants were asked about participation in the study. However, the interview did not start at this time. The informants were asked to participate because they met the study´s inclusion criteria and were included in the order in which they agreed to participate. The contact during the counselling session was probably important in creating trust between the interviewer and the informant and made them open up.

It may be difficult to capture next of kin experiences of what it is like to live close to someone who uses AAS. By using the RLR perspective this has been possible. Thus, despite the inadequate gender distribution, difficulties of capturing experiences and twelve participants, the data collection has nevertheless provided data with rich existential meanings.

All next of kin in this study have in common that they have a close relationship with a person using AAS. Parents, partners, and siblings do not however have the same type of relationship to persons using AAS. These different relationships have made the data more difficult to analyse. It might have been easier if the informants had had the same kind of relation to persons using AAS. On the other hand, the different relationships have made it possible to describe the phenomenon through an increased variety of experiences.

## Conclusion

In this study, next of kin’s lifeworld are described for the very first time in the scientific literature. The lack of research in this field shows the importance of addressing the existing knowledge gaps and of disseminating the results of this study. This research contributes understanding and deep knowledge which are required to be able to meet next of kin in their difficulties and vulnerability when living close to persons with AAS use.

Next of kin are dependent on help and support from other people and authorities. The stress of living close to persons using AAS demands awareness and understanding from others. Involving and taking next of kin into consideration constitute a complex mission that requires understanding, and their lifeworld must be approached with flexibility and empathy. Next of kin may be a resource in many contexts, but not an infinite one. Therefore, society needs to prioritize specific educational efforts for authorities such as healthcare, social services, school, or police and other concerned organizations to increase their knowledge and to be able to face and handle the existing problems. Next of kin are particularly vulnerable because they have minimal opportunities to influence their situation, and health consequences and suffering need to be reduced on an individual level. The results of this study together with educational efforts, increased resources and further research will contribute to increasing the awareness of professionals and function as a support for the existential needs of next of kin. Just as there is support for the families of drug or alcohol abusers or violent individuals, this group of next of kin (both adults and children) must also be given access to programmes that already support other kinship groups.
